# Whole genome sequencing and methylome analysis of the wild guinea pig

**DOI:** 10.1186/1471-2164-15-1036

**Published:** 2014-11-28

**Authors:** Alexandra Weyrich, Tino Schüllermann, Felix Heeger, Marie Jeschek, Camila J Mazzoni, Wei Chen, Kathrin Schumann, Joerns Fickel

**Affiliations:** Leibniz-Institute for Zoo and Wildlife Research (IZW), Alfred-Kowalke-Str 17, D-10315 Berlin, Germany; Berlin Center for Genomics in Biodiversity Research, Koenigin-Luise-Str 6-8, 14195 Berlin, Germany; Max-Delbrueck-Center for Molecular Medicine (MDC), Robert-Roessle-Strasse 10, 13125 Berlin-Buch, Germany

**Keywords:** Methylated DNA-enrichment-bisulfite-sequencing (MEBS), Immunoprecipitation, Methyl-binding domain protein (MBD), Bisulfite, Next-generation-sequencing, Reference sequence, Cavia

## Abstract

**Background:**

DNA methylation is a heritable mechanism that acts in response to environmental changes, lifestyle and diseases by influencing gene expression in eukaryotes. Epigenetic studies of wild organisms are mandatory to understand their role in e.g. adaptational processes in the great variety of ecological niches. However, strategies to address those questions on a methylome scale are widely missing. In this study we present such a strategy and describe a whole genome sequence and methylome analysis of the wild guinea pig.

**Results:**

We generated a full Wild guinea pig (*Cavia aperea*) genome sequence with enhanced coverage of methylated regions, benefiting from the available sequence of the domesticated relative *Cavia porcellus*. This new genome sequence was then used as reference to map the sequence reads of bisulfite treated Wild guinea pig sequencing libraries to investigate DNA-methylation patterns at nucleotide-specific level, by using our here described method, named ‘DNA-enrichment-bisulfite-sequencing’ (MEBS). The results achieved using MEBS matched those of standard methods in other mammalian model species. The technique is cost efficient, and incorporates both methylation enrichment results and a nucleotide-specific resolution even without a whole genome sequence available. Thus MEBS can be easily applied to extend methylation enrichment studies to a nucleotide-specific level.

**Conclusions:**

The approach is suited to study methylomes of not yet sequenced mammals at single nucleotide resolution. The strategy is transferable to other mammalian species by applying the nuclear genome sequence of a close relative. It is therefore of interest for studies on a variety of wild species trying to answer evolutionary, adaptational, ecological or medical questions by epigenetic mechanisms.

**Electronic supplementary material:**

The online version of this article (doi:10.1186/1471-2164-15-1036) contains supplementary material, which is available to authorized users.

## Background

One mechanism species have evolved to adapt to variations on a molecular level is epigenetic modification, such as DNA methylation. DNA methylation is one mechanism that regulates gene expression in eukaryotes [1, 2]. In mammals it is essential for embryonic viability due to its function in developmental processes such as imprinting, X chromosome inactivation, cell differentiation, gene regulation, and transposon silencing [1, 3, 4]. Dysregulated DNA methylation was found in the etiology of many diseases, including cancer [5, 6]. The methylation pattern is mitotically and sometimes even meiotically heritable [7]. Its pattern is stably maintained, but can also be very flexible, as in response to environmental changes. DNA methylation is the enzymatic addition of a -CH_3_ group to the 5’carbon site of cytosines [8] and occurs in mammals mainly at CG dinucleotide sites (CG), of which 70-80% are methylated [9–11]. In rare cases methylation also occurs at CHG and CHH trinucleotide sites (H representing any nucleotide but G; [12, 13]). Clusters of CGs, called CpG islands (CGI) [14, 15] are, however, mostly unmethylated [16]. In the vertebrate genome about 70% of all promoters are associated with CGIs.

In general, methylation of promoters causes transcriptional silencing [17, 18], as they become less accessible to transcription factors. This effect is enhanced by members of the methyl-CpG-binding domain protein family (MBD) whose binding to methylated sites induces conformational changes to the chromatin [19].

Several methods have been developed to study DNA methylation on a genomic scale, each having strengths and weaknesses [20–22]. These methods mostly incorporate next generation sequencing (NGS) and are classified in bisulfite-treatment based methods and enrichment techniques [23].

So far, studies on DNA methylation were mostly restricted to model organisms as these are easier to study because required genomic data is available. Here we present a strategy to study the genome-wide DNA methylation pattern in the Wild guinea pig *Cavia aperea*, a mammal species that has not yet been genomically sequenced. The strategy is based on utilizing the genome sequence of a close relative (here *Cavia porcellus*) and on an enhanced coverage of methylated regions. In the first step, we generated a ‘*C. aperea* reference sequence’ whose coverage was increased specifically for methylated sequences. To further study cytosine methylation we established a method which involves the enrichment of methylated sequences using MBD2, bisulfite treatment, and NGS, which we named ‘methylated DNA-enrichment-bisulfite-sequencing (MEBS)’. The method permits the detection of cytosine methylation at single-base resolution yet at affordable costs. Using MEBS we obtained methylome data, comparable with those obtained in model-mammals [10, 11, 16, 24].

## Results

### Cavia aperea reference sequence

To generate a new reference sequence for *C. aperea*, we first sequenced two pooled Illumina mate-pair (MP) libraries of two individuals (Table 1, library 1–2). The sequences were mapped onto the *C. porcellus* reference sequence (cavPor3), revealing a 4-fold average genomic coverage (number of mapped reads × read length/genome size). To increase coverage at sites of interest, we mapped in a second step also sequences of six methylation site-enriched paired-end (PE) libraries (Table 1, library 3–8) onto *C. porcellus*, thereby enhancing the average coverage of methylated regions to 333×. Thus, by combining these two library types in the mapping process we generated a methylation site enhanced reference sequence of *Cavia aperea* (Table 1). We then annotated 20,653 genes (out of 26,129 genes known for *C. porcellus*) of which 14,003 were protein coding. Additionally, we annotated 22,574 CGIs.

**Table 1:** **Source, details and general results of sequence libraries**

No	Library	Animal ID	Tissue	Read length	Fragment size [bp]	No. of clean reads	Average coverage
1	MP	C. aperea_2	Muscle	100 bp	2200	123,511,268*	4x
2	MP	C. aperea_3	Liver	100 bp	2200		
3	MBD2-PE	C. aperea_1	Testis	100 bp	264-1253	195,674,422	7.3x
4	MBD2-PE	C. aperea_2	Testis	100 bp	249-1326	172,854,726	
5	MBD2-PE	C. aperea_4	Liver	100 bp	203-672	146,575,288	
6	MBD2-PE	C. aperea_5	Liver	100 bp	214-743	144,660,328	
7	MeDIP-PE	C. aperea_1	Testis	100 bp	218-817	87,769,840	0.82x
8	MeDIP-PE	C. aperea_2	Testis	100 bp	219-990	71,262,748	
9	MEBS-PE_1	C. aperea_1	Testis	90 bp	235	119,880,204	1.02x**
10	MEBS-PE_2	C. aperea_2	Testis	90 bp	238	109,353,416	0.66x**

MP = Illumina mate pair library; PE = Illumina paired end library; *MP libraries were pooled before sequencing; Average coverage = No. of mapped reads × read length/*C. porcellus* genome size. **Average coverage = No. of mapped reads × read length/*C. aperea* genome size.

### Methylated DNA (mDNA)- enriched PE libraries

We enriched for methylated DNA (mDNA) from total genomic DNA (gDNA) using either MBD2 (Methyl-binding domain2) protein or an antibody directed against mCs (methylated DNA immunoprecipitation, MeDIP) (Table 1; libraries 3–8). The MBD2 protein bound ~1/10 of the sheared. We prepared four MBD libraries (Table 1; libraries 3–6) and performed sequencing (MBD2-seq). To increase the number of genomic regions covered, we used two different tissues (testis and liver) to profit from cell-specific methylation patterns. Reads covered about 42.6% of the reference sequence of *C. aperea* with an average coverage of 7.3 ×.

To test for reproducibility, we compared genomic sequencing depth among the MBD2-seq reads. We found a slightly stronger correlation of the sequencing depth between the MBD2-seqs that derived from the same tissue (Table 1; library 3 vs. library 4: r = 0.994; library 5 vs. library 6: r = 0.997) than for those derived from different tissue material (library 3 vs. library 5: r = 0.979; library 3 vs. library 6: r = 0.981; library 4 vs. library 5: r = 0.987; library 4 vs. library 6: r = 0.988). The high correlation of coverage indicates high reproducibility, the slightly higher correlation of the same tissue material stems from the tissue-specific methylomes.

As the reads of all four MBD2-PE libraries (Table 1; libraries 3–6) were subsequently combined, we will henceforth generalize them as MBD2-seq. Compared with MBD2 reads, MeDIP reads (Table 1; libraries 7–8) had a lower coverage of just 0.84× refraining us from analyzing them any further. We did, however, incorporate these reads into the final reference sequence to further enhance sequence information.

### MEBS data

**Figure 1:**
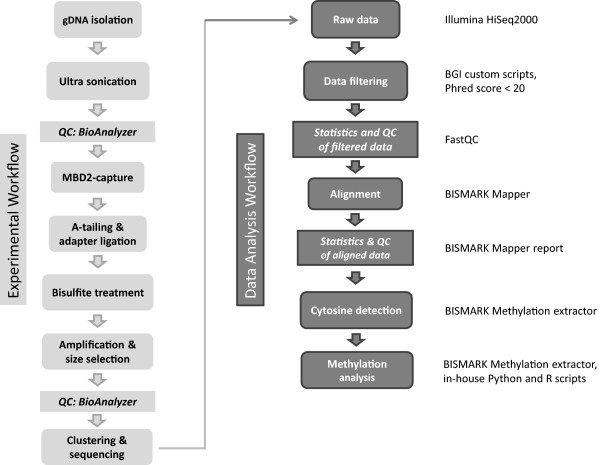
**Flowchart for library preparation and treatment until sequencing (light gray) and bioinformatic analysis (dark gray) and software used for each step.** gDNA = genomic DNA; QC = quality control.

MEBS was performed on two *C. aperea* MBD2-PE libraries (Figure 1; Table 1, libraries 9–10). Here we used testis DNA, but any mammalian tissue material is applicable. For both *C. aperea* libraries, 99% of MEBS reads passed the quality threshold of Q20 and 96% even passed Q30. We used Q20 data for downstream analysis. After filtering and trimming, we successfully obtained 11.9 × 10^7^ and 10.9 × 10^7^ clean reads for the two libraries, respectively (Table 2 and Table 1, libraries 9–10). Procedure reproducibility was shown by a strong positive correlation (r = 0.94) between the fragment coverage of both MEBS libraries as well as between the methylation ratios of all C positions covered in both libraries (r = 0.94). Using MEBS we were able to check for MBD2-binding specificity. Only 0.6% of *C. aperea* reference positions covered by MEBS were not covered by MBD2-seq, while 10.8% were covered by both. MBD2-seqs alone covered 31.8% of all reference positions, while MEBS reads alone covered 11.4% of all reference positions.

**Table 2:** **MEBS output: quality of clean reads and mapping results per library**

Library	Clean reads	Mapped reads	Coverage [%]	Sequencing depth
		[%]		[x-fold]
MEBS-PE_1	119,880,204	29.5	11.21	9.09
MEBS-PE_2	109,353,416	26.4	9.27	7.54

Clean data and mapped reads were based on Q20 values. Coverage refers to the ‘*C. aperea* reference sequence’ (including Ns set as 100%). The sequencing depth was calculated per covered region.

### Reference sequence validation

We generated several reference sequences using different combinations of libraries to investigate the relation between cost and benefit of different library preparation and sequencing methods (see Methods and Additional file 1: Table S1). To check for and ascertain best mapping efficiency, all MEBS reads were mapped to those reference sequences using Bismark (see Additional file 2: Table S2). The highest percentages of mapped MEBS reads for both libraries were obtained using the newly generated ‘*C. aperea* reference sequence’, in spite of its higher N content (undetermined positions in *C. aperea*: 22%; see Additional file 1: Table S1) compared with *C. porcellus* (Ns: 2%).

We also generated a reference from MBD2-seqs without incorporating MP and MeDIP-capture reads. Mapping to this reference resulted in similar results as mapping to the new ‘*C. aperea* reference sequence’ (see Additional file 1: Table S1). Comparing the numbers of MP reads (123,511,268; Table 1) and MBD2-seq reads (329,882,382 for 2 libraries) it became clear that incorporation of MP reads into the reference improved mapping efficiency by only 0.85%, and 1.20%, respectively, for the two MEBS-PE libraries.

These results demonstrate that additional incorporation of non-enriched reads was not mandatory to create the necessary reference sequence and that MBD2-seq reference provided a cost effective alternative for the MEBS workflow.

We also compared the positions of the mapped reads among all reference sequences, to see if mappings to different reference sequences were consistent (see Additional file 2: Table S2). We found that the positions of mapped reads were highly consistent, regardless of the reference sequence the read was mapped to. The reads of the two MEBS-PE libraries mapped with 96.79% and 94.80%, respectively to similar positions in the *C. aperea* and *C. porcellus* reference sequence, indicating a correct placement of the reads.

### DNA methylation analysis

For mC determination we focused on reads that mapped uniquely onto the ‘*C. aperea* reference sequence’. Unique mapping rates were 29.88% and 26.38%, respectively for the two MEBS-PE libraries (Table 2; see Additional file 1: Table S1), whereas 11% and 12% of the reads did not map unambiguously. Reads of the two MEBS-PE libraries (MEBS-PE_1 and _2) covered 11% and 9% of the ‘*C. aperea* reference sequence’ (Table 2).

**Figure 2:**
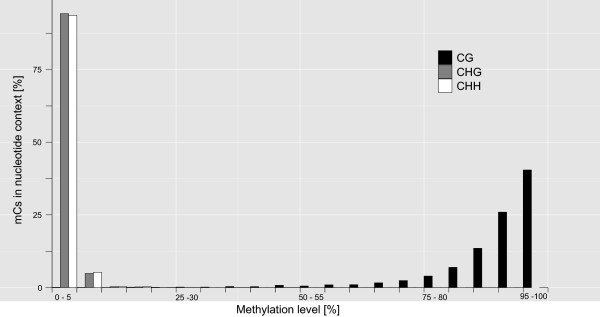
**Proportion of mCs at CG, CHG and CHH sites detected in MEBS reads.** The histogram shows the cytosine methylation level per genomic context CG, CHG and CHH, whereby H represents any nucleotide but G. Highest methylation levels were present in CG dinucleotides (applying average data of both MEBS libraries). CHH and CHG were methylated at very low levels.

We explored the degree of methylation and the locations of mCs. According to the ratio of CGs, CHGs, and CHHs, the efficiency of bisulfite treatment was ~99% for both MEBS-PE libraries. In both libraries ~18% of the detected cytosines were methylated. Out of those ~90% were within CG dinucleotides (Table 3). Four mCs per mapped read occurred most often (see Additional file 3: Figure S1), while less than 1% of cytosines in CHH and CHG (H = either A, T or C) were methylated, respectively (Table 3 and Figure 2).

**Table 3:** **Total number of mCs and their methylation levels [%] in dependence on nucleotide context**

Library	mCG	mCHG	mCHH
MEBS-PE_1	130,680,264	1,801,270	3,543,614
	90.76%	0.79%	0.91%
MEBS-PE_2	102,257,162	1,290,501	2,858,916
	90.60%	0.80%	0.93%

Presence of mC depending on the immediate following nucleotides in which H represents non-G bases (H = A, T or C). The 100% reference value differs per column as percentages were calculated for mCs being part of mCGs according to the following equation: (Ʃ mCG/Ʃ (mCG + CG)) × 100 = % mCs of CGs. Accordingly, calculations were carried out for mCs being part of mCHGs and mCHHs.

The method also allows to identify if non-methylated regions had also been (unspecifically) captured by MBD2. In only 1.26% of all uniquely mapped MEBS-PE reads, all cytosines were converted by bisulfite treatment (and thus had not been methylated before), indicating a high specificity of MBD2 to mDNA.

**Figure 3:**
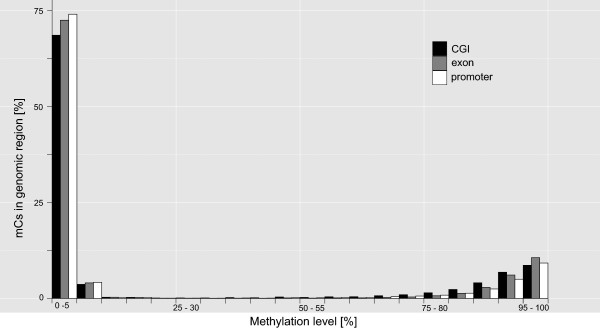
**Proportion of cytosines being methylated in genomic regions of interest.** Cytosine methylation localized in CGIs, exons and promoters. All regions investigated were either low or highly methylated (applying average data of both MEBS libraries). Promoters were defined as 2 kb upstream the TSS, here without CGIs.

**Figure 4:**
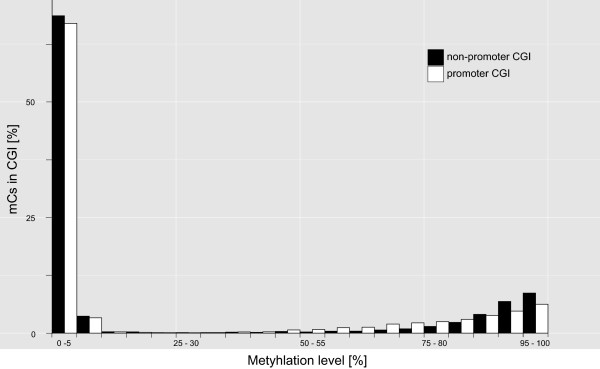
**Proportion of cytosines methylated in promoter CGIs and non-promoter CGIs.** After dividing CGIs in CGIs inside and outside promoters (promoter CGIs and non-promoter CGIs) it became obvious that cytosine methylation was more prominent in non-promoter CGIs (applying average data of both MEBS libraries).

We also investigated genomic regions of interest for their percentage of mCs. CGIs (inside and outside of promoters), exons and promoters (without CGIs) displayed similar methylation levels, which were either very high (75-100%) or very low (0-4%) (Figure 3). Intermediate methylation levels were nearly absent. As CGIs are a main target of DNA methyltransferases we also separately analysed the percentage of mCs in CGIs inside and outside of promoter regions and found no obvious difference in the level of methylation between them (Figure 4). Out of the 22,574 CGIs annotated in total, only 538 (2.4%) were located in promoter regions. We also found only a few CGIs in close proximity to promoters (see Additional file 4: Figure S2). The vast majority (78%) of CGIs was at least 10,000 base pairs away from a promoter. Thus, the low amount of CGIs associated with promoters is not an effect of the definition of promoter regions as 2 kb upstream of the transcription start site (TSS).

### Gene selection for methylation analysis

**Figure 5:**
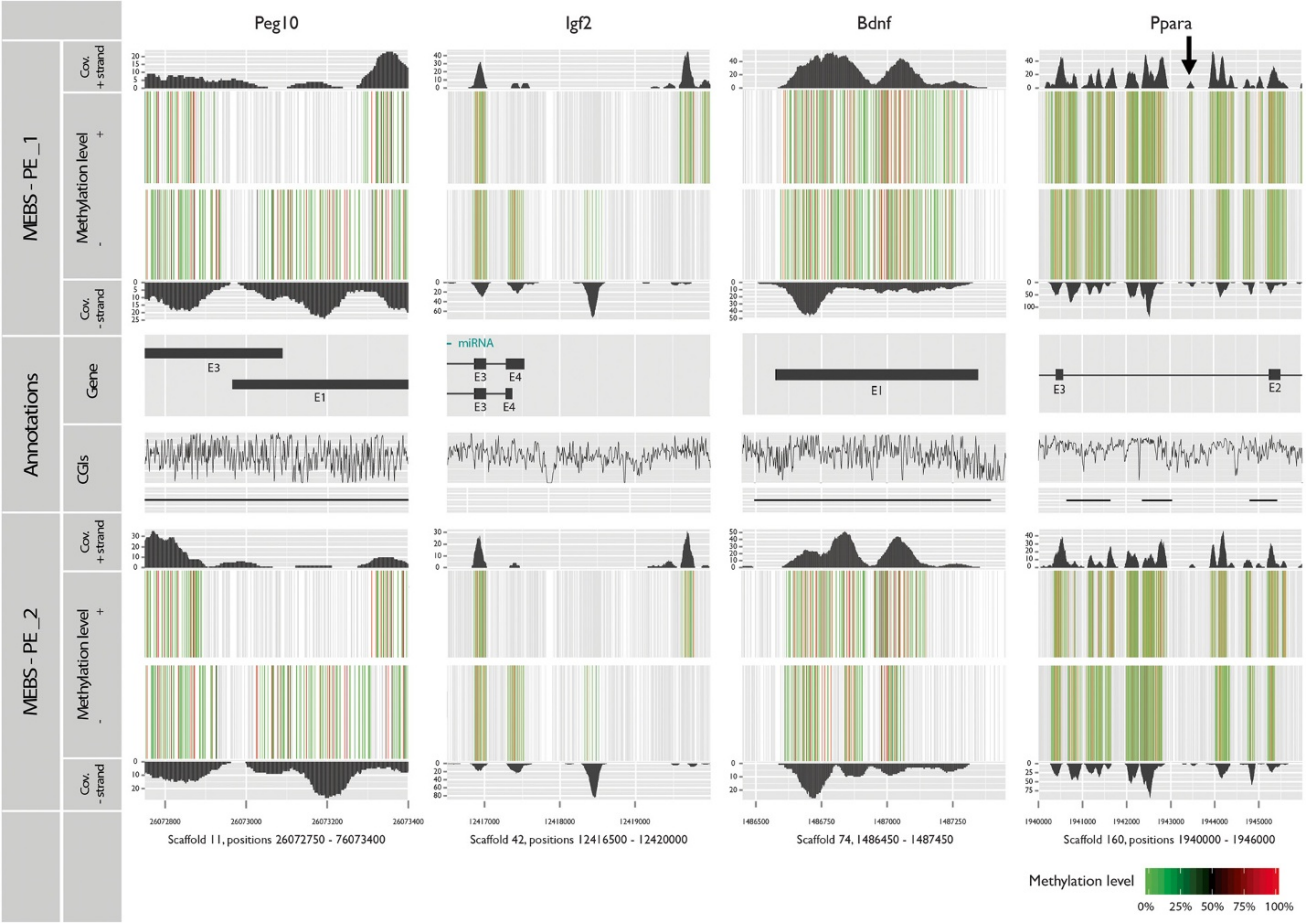
**Genes selected for detailed methylation level analysis.** Methylation levels were visualized for four methylated genes (columns) in both libraries (upper row: MEBS-PE_1, lower row: MEBS-PE_2). Those genes were genes known to be methylation-regulated such as the *Brain-derived neurotrophic factor (Bdnf)*, and *Peroxisomal proliferator-activated receptor alpha (Ppara)* as well as the two imprinted genes *Paternally expressed imprinted gene 10 (Peg10)* and *Insulin-like growth factor 2 (Igf2)*. Low (0-40% in green), intermediate (40-60% in black) and high methylation levels (60-100% in red) were shown for both strands, according to their coverage. Annotated positions (see Scaffold row) for genes (exon, introns) and CGI are depicted in the middle row. The CGIs row is divided in two sections: the lower section contained CGIs (black lines) which were calculated by the percentage of CG dinucleotides, depicted in the upper section as frequency graph. Dense high peaks represented high CG frequency, indicating CGIs. Genes were annotated as exons (thick bars) and introns (thin bars). In *Igf2*, the co-located and co-regulated miRNA (blue bar) was shown above the second intron. Inter-library differences were minor, but can be seen e.g. for Ppara (arrow) where methylation levels were detected only in MEBS-PE_1.

As proof of principle we looked for paternally expressed imprinted genes in the reads of both MEBS-PE libraries. As these genes are known to be regulated by methylation we expected them to be present in the libraries. We also expected both libraries to show similar results because the libraries were from the same type of organ of two coeval male Wild guinea pigs, housed under the same conditions and thus serving as biological replicates. As expected, the MEBS-PE libraries contained reads of numerous imprinted genes (see Additional file 5: Table S3) of which two were analyzed in greater detail: the *Insulin-like growth factor 2* (*Igf2*) and the *Paternally expressed gene 10* (*Peg10*; Figure 5). We also looked beyond the paternally imprinted genes for additional, strongly methylated genes (see Additional file 5: Table S3) of which we chose two to be displayed in greater detail: the *Brain-derived neurotrophic factor gene (Bdnf)* and the *Peroxisomal proliferator-activated receptor alpha* (*Ppara*; Figure 5). As expected the methylation patterns of both libraries showed high correlations at these loci (*Igf2*: r = 0.99, *Peg10*: r = 0.97, *Bdnf*: r = 0.98, *Ppara*: r = 0.98) confirming the method’s credibility and reproducibility.

## Discussion

### Main goal

The growing interest in DNA methylation as a main epigenetic mechanism involved in the expression of numerous phenotypic traits has also increased the demand for technologies for its characterization. As wild species carry important information of evolutionary adaptational processes, but mostly have not yet been sequenced, technical strategies are required to nevertheless study DNA methylation in these species. Here we report an efficient strategy to study genome-wide cytosine methylation patterns at nucleotide-specific resolution in the Wild guinea pig *Cavia aperea*. This was done by the generation of a ‘*C. aperea* reference sequence’ and the incorporation of mDNA-enriched sequences to increase coverage of those specific sites for methylome studies. With such reference, we set the base to perform bisulfite sequencing of mC-enriched regions, using the methodical approach presented here, MEBS. MEBS is the succession of mDNA-enrichment, bisulfite treatment, and next-generation sequencing (mDNA-enrichment-bisulfite-sequencing = MEBS). We showed that combining these methods is an efficient way to study methylation patterns.

### Cytosine methylation

In total about 20% of all covered cytosines of the *C. aperea* genome were methylated in testis cells. As shown for other mammals and thus expected, these mCs were predominantly located within CG dinucleotides of which ~90% were at a methylated state. Compared with the 70-80% of CG dinucleotides being methylated in humans [11], this proportion is higher, which likely stems from the usage of a methylation site enriched sequence causing a slightly biased over-all methylation rate. In *C. aperea* CHG and CHH sites were rarely methylated. It is known from humans, that CHG and CHH methylation disappears with the induction of cell differentiation in embryonic and other stem cells [10]. About 25% of mCs are in a non-CG context, and become *de*methylated upon induced stem cell differentiation and *re*methylated in induced pluripotent cells. An exception was found in human and mice brain cells, where non-CG methylation is highly conserved in regions with low CpG-density, and likewise to mCG represses gene transcription [25, 26].

### Methylated CGs in genomic regions

In the wild guinea pig, we found CGs in exons to be highly methylated (Figure 3). This pattern was expected as it is strongly conserved among plants and mammals [27].

When considering the difference in genome size, the number of CGIs in *C. aperea* (=22,574; genome size 2.7 Gb, and 2.12 Gb without Ns) was about 40% smaller than the number of CGIs in a human genome (=37,531; genome size 3.3 Gb, 2.85 Gb without Ns [28]). In the vertebrate genome about 70% of all promoters are associated with CGIs [16]. Among human and mouse about 40% of promoter CGIs are methylated at orthologous genomic regions [29, 30]. Although in *C. aperea* CGIs and promoters had similar methylation densities, most mCs were found in non-promoter CGIs (Figure 4). Those non-promoter CGIs may either belong to still unknown gene promoters [29], may represent additional regulatory units of the genome, or are part of retrotransposons like short interspersed nuclear elements (SINE; e.g. Alu-elements), which in humans represent 10% of the genome [31].

Most wild guinea pig promoters showed low methylation levels. The majority of the regions analyzed were either hypo (0-4% of all mCs) or hypermethylated (75-100%; Figures 3 and 4), potentially indicating a regulatory function of methylation in gene expression. This conclusion, however, needs to be viewed cautiously, as the method itself is targeted at sequencing fragments containing mCs, thereby influencing the detection ratio of hypo-, meso, and hypermethylated reads. The detection of hypomethylated fragments at high frequency, however, gives our general conclusion plausibility.

By investigating specific genes in two biological replicates (Figure 5), we observed similar and strongly correlated methylation patterns, indicating that the detected methylation patterns were not arbitrary but rather of general importance.

### Advantages of the MEBS method

The main advantage is that it enables the easy incorporation of methyl-enrichment results to track methylation with nucleotide-specific resolution without whole genome sequences available.

By combining the data from the methylation-site enriched libraries (MBD-seq or MeDIP-seq data) with the data obtained after bisulfite treatment, the already existing enrichment reads can be used to create a mDNA-enriched sequence that the MEBS-PE reads can be mapped onto. Methylation ratios can be easily calculated (Figure 5) and non-methylated “false positive” reads that were unspecifically captured by the MBD2 protein can be identified as such.

It is possible to perform MEBS with any mDNA enrichment kit, as well as with single and paired end (PE) library preparation kits. We used PE sequencing, because it provides sequence information from the forward and the reverse strand. The two matched reads gave a greater genomic range (fragment size), which can be mapped more accurately.

Using MEBS, different methylation states can be studied easily, such as cell type-specific methylation pattern and/or effects of environmental changes on methylation patterns. After identification of loci that are methylated in one state but not the other using the enriched methylated fragments, cytosine-specific methylation at those loci can be measured, permitting to determine hypo and hypermethylation, and allowing to detect minor changes among states within captured loci.

### Reference sequences

In the present set-up we benefited from the existing genome sequence of a close species, the domestic guinea pig (*C. porcellus*), which diverged from the wild guinea pig (*C. aperea*) just ~7,000 years ago). The high similarity between these species certainly facilitated the establishment of MEBS. However, with the speedily growing pool of high quality mammalian genome sequences, the method should be transferrable to numerous other mammalian species.

Among all our mapping approaches onto different references (see Additional file 1: Table S1), the ‘*C. aperea* reference sequence’ yielded the best mapping results for the reads of the two MEBS-PE libraries. Despite the fact that the newly generated ‘*C. aperea* reference sequence’ had 22% Ns instead of only 2% Ns in the *C. porcellus* reference sequence, we still achieved a mapping rate improvement of 2.57% for MEBS-PE_1 (Table 1, library 9) and of 6.03% for MEBS-PE_2 (Table 1, library 10). The preparation of a mDNA-enriched sequence is a cost-effective, yet accurate way to produce a reference sequence for DNA methylation studies.

Interestingly, the reference sequence generated by MBD2-seq only, still gave a better mapping result than the *C. porcellus* reference sequence we used as initial reference sequence (see Additional file 1: Table S1). Compared with the low coverage of MP reads (4×) and the even lower coverage of MeDIP-capture reads (0.84×), which only led to a low increase in mapping efficiency, the best results were obtained using MBD2-captured reads (coverage: 7.3×) and MEBS-PE reads of the same sample material. Therefore, the generation of a MBD2-seq is efficient and additional sequencing is not mandatory to obtain reliable results for the MEBS pipeline. This aspect could potentially reduce a study’s processing time and experimental costs. It should be noted that although incorporation of MP reads had (in our case) no effect on the mapping results, it decreased the number of Ns in the newly generated ‘*C. aperea* reference sequence’.

### Dependence on MBD2 protein

A comparison of four commercially available MBD-based enrichment kits [32] found that the MBD2 protein based MethylMiner™ Kit (Invitrogen) gives the highest DNA yield, as well as the most “non-duplicated uniquely mapped fragments” [32–38]. The same study, however, also stated that MethylMiner™ also captures unmethylated sequences, wherefore it ranked last in sensitivity and specificity [32]. Although we did not test other capture kits, our results clearly showed that MethylMiner™ kit captured fragments even with as little as just a single mC present (Figures 2, 3, 4 and 5 and see Additional file 3: Figure S1), demonstrating a very high sensitivity to methylated sites. In our study, specificity was very high as well, as only a total of ~1.26% of captured MEBS fragments were unmethylated.

The high capture yield of the MethylMiner™ kit also permitted capturing of the required amounts of DNA recommended for bisulfite treatment and NGS from a small amount of sample material.

The different sensitivities of the kits [32] stem most likely from the MBD protein included within the kit. The MBD protein family in mammals consists of MBD1, MBD2, MBD3, MBD4 and the methyl-CpG-binding protein MeCP2 [19, 33]. In general, MBD1, MBD2, MBD4 as well as MeCP2 proteins mainly bind to methyl-dense CG-rich positions. But MBD2 and MBD4 may also bind to unmethylated targets [2, 34, 39] if these regions are low in CG density and DNA methylation, but co-located with transcriptionally active histone modification sites, such as H3K4me1 and H3K27ac. MBD2 also shows binding-specificity to active promoters and enhancer elements [34] where it induces conformational changes to the chromatin [19]. Those findings imply an additional function of MBD2 within the DNA methylation pathway. In our case, the broader spectrum of sites that MBD2 recognizes provided the advantage of a broader spectrum of sequence information.

### Comparison with other methods

Bisulfite sequencing of whole genomes remains the “gold standard” but is still very costly, creates huge amounts of data, which, as they consist of mainly non-methylated sequences, are of less interest when addressing the methylome. Both reasons are prohibitive for greater sample numbers. MEBS reduces cost and output of (excessive and uncalled-for) sequencing data while still covering the methylated genomic positions of interest.

MEBS also offers an alternative to the Reduced Representation Bisulfite Sequencing (RRBS) method. Although RRBS also reduces the output of genomic sequences and includes bisulfite treatment [9], it relies on the application of restriction enzymes and fragments are size-selected according to their suitability for sequencing instead for biological reasons, creating high amounts of products to be excluded. Nevertheless, RRBS is an efficient method to address DNA methylation on a genomic scale [9]. Depending on the restriction enzyme(s) used in RRBS, the amount of DNA to be sequenced is reduced to ~1-9% of the genome [9]. Our MEBS method, however, covered between 9% (MEBS-PE_2) and 11% of the genome of *C. aperea* (MEBS-PE_1). Furthermore, while the use of restriction enzymes in RRBS (e.g. MspI) enriches fragments with high CG content such as CGIs, it widely neglects other methylome relevant regions. MEBS selects methylated regions regardless of their origin or their surrounding nucleic sequences. Therefore we were able to address genes as well as promoter regions with diverse content of mCs (Figure 3). Additionally, RRBS protocols are currently only established for single-end library sequencing. We used paired-end sequencing, which in general increases the coverage and thus accuracy of each sequenced fragment.

Parallel to the establishment of our method, a similar approach called “MethylCap-seq” was published [40]. Despite their similar experimental setups, both methods differ in the MBD protein used, and more importantly, in their sequencing approach. Like RRBS, MethylCap-seq is based on single-end library sequencing with a read length of 36 bp, whereas our method is based on 90 bp long reads. Using paired-end sequencing we have a ~3× larger final fragment size of 134 bp.

But why not use commercially available array systems? These arrays limit the results to predefined regions such as CGIs or promoters and exclude other regions [18]. Furthermore, such arrays depend on known sequence information for the species of interest or for closely related species in conjunction with a custom-array design. The first option would lead to a loss of non-orthologous sequences, and simultaneously might cause false positive results by mismatch-hybridization. The latter is time-consuming, costly and restrictive.

## Conclusions

In this study, we describe 1) the generation of a whole genome sequence of the Wild guinea pig by applying the genome of its sister species and 2) MEBS as an efficient and reproducible method for DNA methylation analysis. The combined strategy can be applied to increase the understanding of epigenetic modification, such as gene × environment interactions in wild life.

## Methods

### Sample material and DNA isolation

All husbandry and experimental procedures were approved by the German committee of Animal Welfare in Research (permit no. V3-2347-35-2011). Wild guinea pigs (*Cavia aperea*) were obtained from F. Trillmich (University of Bielefeld) and housed at the IZW field station in Niederfinow, Germany. Sample material of muscle, liver and testis samples were taken from five male guinea pigs (Table 1), shock frozen in liquid nitrogen and stored at −80°C. DNA was isolated using the First-DNA all-tissue Kit (GEN-IAL GmbH, Troisdorf, Germany).

### Mate pair and mDNA enriched paired-end library preparation and sequencing

For whole genome sequencing, we prepared two Illumina 2 kb Mate pair (MP) libraries from both a muscle and a liver tissue sample of two wild guinea pigs (Table 1) in accordance to the sample preparation guide (Part #15008135 Rev. A.; v2). mDNA for methylome sequencing was obtained from liver and testis samples via MeDIP and MBD2-capture (see below) and used to prepare six paired-end (PE) libraries applying the Illumina DNA TruSeq Kit V3 (Illumina, Munich, Germany). The eight libraries (Table 1, libraries 1–8) were sequenced on an Illumina HiSeq2000 at the Max Delbrueck Center (MDC, Berlin, Germany).

### MEBS sample and library preparation

Two MEBS libraries were generated from testis tissue of two coeval males, housed under similar conditions (Table 2; for workflow see Figure 1). We used 10-12 μg genomic DNA (gDNA) per animal (1 μg per shearing tube) to be sheared to 200-400 bp fragments on a S220 Focused-ultrasonicator (Covaris, Woburn, Massachusetts, USA). Fragment length was verified on a 2100 Bioanalyzer (Agilent Technologies, Waldbronn, Germany) using a DNA 1000 Kit (Agilent). Small fragments (<100 bp) were removed using AMPure Beads XP (Beckmann Coulter GmbH, Krefeld, Germany). Methylated DNA (mDNA) fragments were enriched by capture using human MBD2 protein following the instructions of the MethylMiner™ Methylated DNA Enrichment Kit (Invitrogen, Karlsruhe, Germany) with one final elution step. The initial 4 μg of genomic DNA (gDNA) per reaction required by the Kit yielded 300-400 ng (~10%) of mDNA, measured with a Quant-iT™ PicoGreen® dsDNA Assay Kit (Invitrogen, Karlsruhe, Germany). An external control system (positive and negative control DNA of the MethylMiner™ Kit) indicated mDNA-specific binding of MBD2. Enrichments were run in triplicates to gain at least 1 μg of mDNA fragments for each library preparation, using the Illumina DNA TruSeq Kit V3 (Munich, Germany). The mDNA of each sample was end-repaired, and a 3’-dA overhang was added. Next, methylated adapters (part of the Illumina DNA TruSeq Kit V3, Munich, Germany) were ligated and the libraries (~200 ng DNA) were bisulfite-treated (EZ DNA Methylation-Gold™ Kit, Zymo Research, USA). The use of methylated adapters is crucial, as bisulfite induced adapter conversion would prevent binding to the Illumina flow cell. After desalting and on-gel size-selection (40-300 bp), libraries were amplified by PCR using Illumina standard adapter primers. The libraries were then again size-selected for fragments of 80-200 bp length. Library quality control was carried out by qPCR on a BioAnalyzer (Agilent) using a High sensitivity assay kit. PE sequencing of 90 bp reads was performed on an Illumina HiSeq2000 (BGI Genomics, HongKong, China).

### MEBS data filtering & mapping

To achieve good mapping results, low quality reads were removed from the dataset. We checked the read quality by FastQC (V. 0.10.1). Low quality reads were defined as reads containing (i) adaptor sequences, (ii) more than 10% of uncalled nucleotides or (iii) more than 10% of nucleotides with a Phred + 33 quality score <20 (see flowchart Figure 1) (custom-made script).

We used Bismark (v.0.7.12;[35]) to map the bisulfite converted sequencing reads (settings: mismatches ≤2, fragment size 600 bp, directional reads). Ambiguously mapped reads were discarded. From the mapped reads we calculated the average coverage as the number of mapped reads × read length/genome size.

### Generating a reference sequence

Ssaha2 (v.2.5.5; [36]) was used to map the MP and mDNA-enriched reads (not yet bisulfite-treated) onto the *C. porcellus* reference sequence cavPor3 (http://www.ensembl.org/Cavia_porcellus/Info/Index?db=core). In order to preserve as many differences as possible between C. aperea and C. porcellus, we allowed a high error rate of 20% per read. The maximum gap size allowed for each library was set according to the specific fragment size of each library.

All resulting sequence alignment/map (SAM) files were merged using SAMtools (v. 0.1.17; [37]) in order to create a Mpileup file, with *C. porcellus* as reference sequence. The Mpileup file provided position-specific information about the mapping results which we applied to create the (new) ‘*C. aperea* reference sequence’ (in-house Python scripts). We considered positions with a coverage of <4 as not sufficiently covered (indicated by N), whereas for positions with a coverage of ≥4, we selected the base confirmed by most of the reads for the new reference. If two or more bases had the same maximum frequency, one of them was chosen randomly. Insertions and deletions were incorporated in the same way.

### Impact of reference sequence mapping

To evaluate the impact the choice of reference sequence has on the mapping result, we remapped all reads obtained after bisulfite treatment onto the original *C. porcellus* reference sequence. Additionally, we created a MBD2-seq (MBD2-seq-ref) including only the paired end reads generated after MBD2-capture. All mappings were performed with Bismark (v.0.7.12; [35]) applying the parameters described above.

To account for indels, we also considered “shifted” positions as evidence that reads mapped to the same position when comparing two references (see Additional file 2: Table S2). Mapped reads therefore may shift in their positions on the different reference sequences. We defined reads as being mapped to shifted positions, if their mapping position in reference 1 was at least within a ±10% range of the mapping position in reference 2 (read position in ref.1 +/− 10% = read position in ref. 2, see Additional file 5: Table S3).

### Reference sequence annotation

Existing annotations from the *C. porcellus* reference (http://www.ensembl.org/Cavia_porcellus/Info/Index?db=core) could be transferred to the ‘*C. aperea* reference sequence’*,* because the scaffold structure was kept consistent. We accounted for indels among *C. porcellus* and *C. aperea* genome sequences (see Additional file 2: Table S2). In addition to the existing *C. porcellus* annotations, positions of transcription start sites (TSS) of *C. porcellus* were downloaded and adopted from BioMart[38]. Promoter regions were defined as 2 kb upstream of TSS and annotated accordingly. CGIs were annotated directly into the ‘*C. aperea* reference sequence’, using the Perl script by Takai& Jones [31] with default parameters.

### Methylation data analysis

The ‘methylation extractor’ feature of Bismark was used to determine the methylation ratio for each cytosine (including CGs, CHGs and CHHs) in the ‘*C. aperea* reference sequence’. The parameter ‘no overlap’ was specified to avoid double counting of methylated cytosines (mC) when reads of the same fragment overlapped. The Bismark ‘methylation extractor’ report was further used for statistical analysis and visualization of selected gene regions. The overall methylation ratio of a given C in the genome was calculated as the proportion of Cs being methylated in all reads mapping to this position. The proportion of mCs in the genome was determined according to the equation: (∑ mC/∑ mC + C) × 100 = % mCs of all Cs. Proportions of mCs approximate to certain nucleotides were calculated as shown below (Table 3).

We also investigated the number of mCs per fragment (approximated by the number of mCs per read) using in-house Python scripts, to evaluate MBD2-capture efficiency.

Results for selected genomic regions were visualized using the two packages Ggplot2 (Wickham 2009) and Ggbio[41] in an R environment (v.2.15.1; R Development Core Team 2011).

### Availability of supporting data

The new ’*C. aperea* reference sequence’ with increased coverage of methylated regions was submitted to the NCBI GenBank with slight changes due to NCBI guidelines for whole genome sequencing (http://www.ncbi.nlm.nih.gov/biosample/2252454; Acc.No. AVPZ00000001-AVPZ00003131). In addition we published the unchanged sequence on the Zoo and Wildlife Institute webpage http://www.izw-berlin.de, Link ftp://62.141.164.1; user: izw_ftp; password: c.aperea (ca. 611 MB). All NGS library reads are accessible one year after publication of the present manuscript at NCBI-SRA database (ProjectID: PRJNA212237).

## Electronic supplementary material

Additional file 1: Table S1: Proportion of undetermined nucleotides (Ns) per reference sequence. Word document, named: Weyrich_BMC_AdditionalFiles_2014-11-03_resubmission. (DOC 50 KB)

Additional file 2: Table S2: Reference sequence and mapping efficiency of the two MEBS-PE libraries at certain regions. Word document, named: Weyrich_BMC_AdditionalFiles_2014-11-03_resubmission. (DOC 30 KB)

Additional file 3: Figure S1: Number of methylated Cs in fragments. Word document, named: Weyrich_BMC_AdditionalFiles_2014-11-03_resubmission. (DOC 106 KB)

Additional file 4: Figure S2: Spatial distance of CGIs to closest promoter regions. Word document, named: Weyrich_BMC_AdditionalFiles_2014-11-03_resubmission. (DOC 46 KB)

Additional file 5: Table S3: Selection of imprinted genes and of non-imprinted genes regulated by methylation found by MEBS. Word document, named: Weyrich_BMC_AdditionalFiles_2014-11-03_resubmission. (DOC 41 KB)

## Abbreviations

CpG: Cytosine-phosphate-guanine
CHG: Cytosine proximate to any other nucleotide but G, followed by G
CHH: Cytosine proximate to any other nucleotide
CGI: CpG island
PE: Paired-end
MP: MatePair library
MBD2: Methyl-binding domain 2
MBD-seq: Sequencing of MBD-captured fragments
MeDIP: Methylated DNA immunoprecipitation
MeDIP-seq: Sequencing of MeDIP-captured fragments
TSS: Transcription start site
gDNA: Genomic DNA
mDNA: Methylated DNA
MEBS: mDNA-enrichment-bisulfite-sequencing
NGS: Next-generation sequencing.
