# Supplementary material for: Whole genome sequencing and methylome analysis of the wild guinea pig
Source: BMC Genomics. 2014 Nov 28;15(1):1036. doi: 10.1186/1471-2164-15-1036 (PMC4302102; doi:10.1186/1471-2164-15-1036)
Supplement: Supplementary file 1 — Additional file 1: Table S1: Proportion of undetermined nucleotides (Ns) per reference sequence. Word document, named: Weyrich_BMC_AdditionalFiles_2014-11-03_resubmission. (DOC 50 KB) [file 12864_2014_6847_MOESM1_ESM.doc]

Additional file 1: Table S1 - Proportion of undetermined nucleotides (Ns) per reference sequence

| **Reference sequence** | **Sequence length [bp]** | **Number of Ns** | **% Ns** | **Mapping rate* MEBS-PE_1 (Uniquely mapped reads)** | **Mapping rate* MEBS-PE_2 (Uniquely mapped reads)** |
| --- | --- | --- | --- | --- | --- |
| *C. porcellus* | 2,723,219,641 | 59,400,948 | 2 | 27.3% (32,745,474) | 20.08% (21,969,004) |
| *C. aperea* | 2,721,439,169 | 601,367,320 | 22 | 29.88% (35,824,594) | 26.38% (28,855,496) |
| MBD2-seq-ref | 2,721,783,340 | 1,451,960,040 | 53 | 29.03% (33,852,254) | 23.81% (26,047,740) |

*using the Bismark mapper, stringency: max. 2 mismatches

From left to right, the number and percentage of undetermined nucleotides (Ns) in each reference sequence (*C. porcellus*, *C. aperea* and MBD2-seq generated reference sequence (MBD2-seq-ref) is shown, followed by the test of mapping efficiencies for MEBS reads when using the different reference sequences. The number of uniquely mapped reads and mapping rates were listed according to the applied reference genome. Reference sequences were the publicly available *Cavia porcellus* reference sequence (*C. porcellus;* <http://www.ensembl.org/Cavia_porcellus/Info/Index?db=core>), our in-house generated *Cavia aperea* reference sequence incorporating Mate pair (MP) reads, MBD2-seq and MeDIP-seq reads (*C. aperea;* <http://www.ncbi.nlm.nih.gov/biosample/2252454>), as well as a reference sequence generated only by MBD2-seq reads (MBD2-seq-ref).
