# Supplementary material for: Whole genome sequencing and methylome analysis of the wild guinea pig
Source: BMC Genomics. 2014 Nov 28;15(1):1036. doi: 10.1186/1471-2164-15-1036 (PMC4302102; doi:10.1186/1471-2164-15-1036)
Supplement: Supplementary file 2 — Additional file 2: Table S2: Reference sequence and mapping efficiency of the two MEBS-PE libraries at certain regions. Word document, named: Weyrich_BMC_AdditionalFiles_2014-11-03_resubmission. (DOC 30 KB) [file 12864_2014_6847_MOESM2_ESM.doc]

Additional file 2: Table S2 - Reference sequence and mapping efficiency of the two MEBS-PE libraries at certain regions

| **Ref. seq 1 vs. Ref. seq 2** | **MEBS-PE_1/ MEBS-PE_2 libraries** | | |
| --- | --- | --- | --- |
|  | **Percentage of same position in both ref. seqs** | **Percentage of different positions in both ref. seqs** | **Percentage when positions were shifted*** |
| *C.aperea* vs. *C.porcellus* | 0.86 / 0.75 | 2.35 / 4.45 | 96.79 / 94.80 |
| MBD2-seq-ref vs. *C.aperea* | 2.53 / 2.52 | 2.91 / 5.26 | 94.56 / 92.22 |
| MBD2-seq-ref vs. *C.porcellus* | 0.89 / 0.79 | 1.72 / 2.98 | 97.38 / 96.23 |
| MBD2-seq-ref vs. MP-ref | 0.03 / 0.02 | 84.82 / 87.69 | 15.15 / 12.30 |

Ref. seq = reference sequence; *We defined reads as being mapped to shifted positions, if their mapping position in reference 1 was at least within a ± 10 % range of the mapping position in reference 2 (read position in ref.1 +/- 10% = read position in ref. 2).
