# Supplementary material for: Whole genome sequencing and methylome analysis of the wild guinea pig
Source: BMC Genomics. 2014 Nov 28;15(1):1036. doi: 10.1186/1471-2164-15-1036 (PMC4302102; doi:10.1186/1471-2164-15-1036)
Supplement: Supplementary file 3 — Additional file 3: Figure S1: Number of methylated Cs in fragments. Word document, named: Weyrich_BMC_AdditionalFiles_2014-11-03_resubmission. (DOC 106 KB) [file 12864_2014_6847_MOESM3_ESM.doc]

**Additional file 3: Fig. S1 - Number of methylated Cs in fragments**


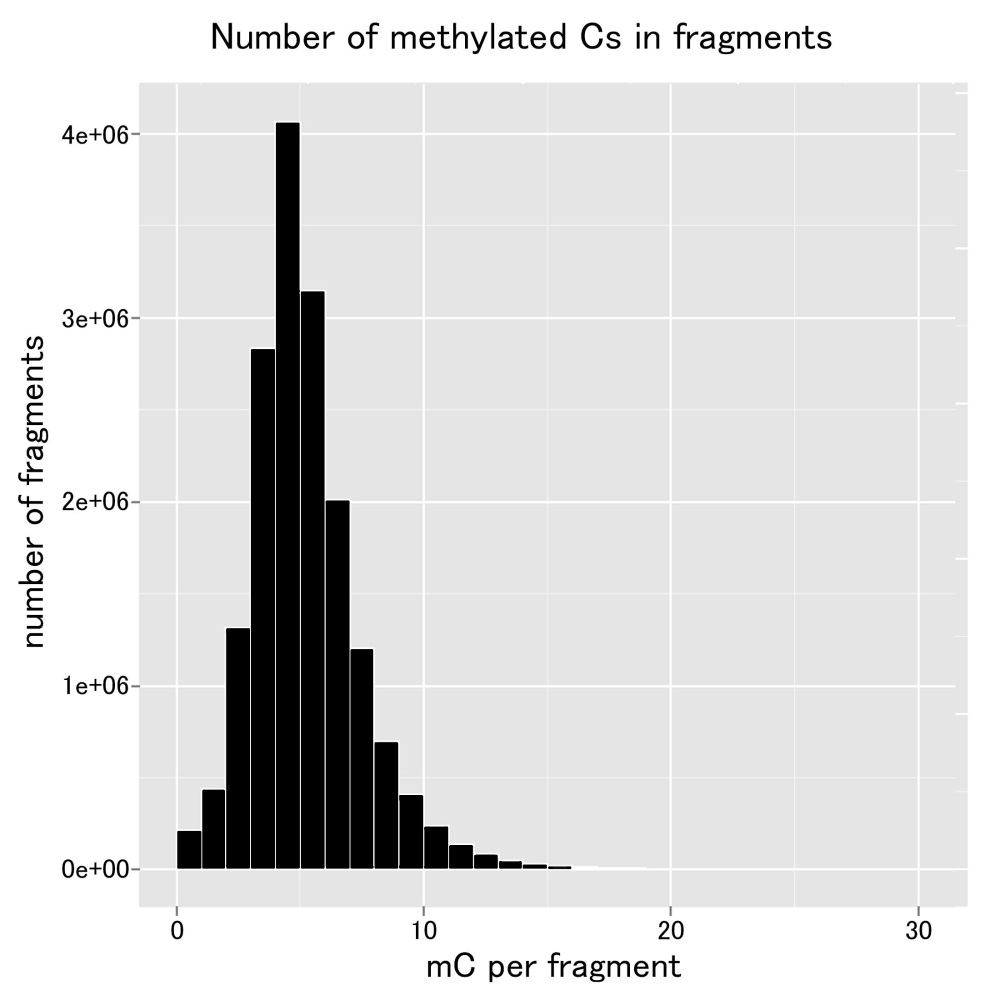


The histogram shows the amount of mCs per MEBS fragment (combined paired-end reads). The highest number of fragments carried four mCs.
