# Supplementary material for: Whole genome sequencing and methylome analysis of the wild guinea pig
Source: BMC Genomics. 2014 Nov 28;15(1):1036. doi: 10.1186/1471-2164-15-1036 (PMC4302102; doi:10.1186/1471-2164-15-1036)
Supplement: Supplementary file 4 — Additional file 4: Figure S2: Spatial distance of CGIs to closest promoter regions. Word document, named: Weyrich_BMC_AdditionalFiles_2014-11-03_resubmission. (DOC 46 KB) [file 12864_2014_6847_MOESM4_ESM.doc]

**Additional file 4: Fig. S2 - Spatial distance of CGIs to closest promoter regions**


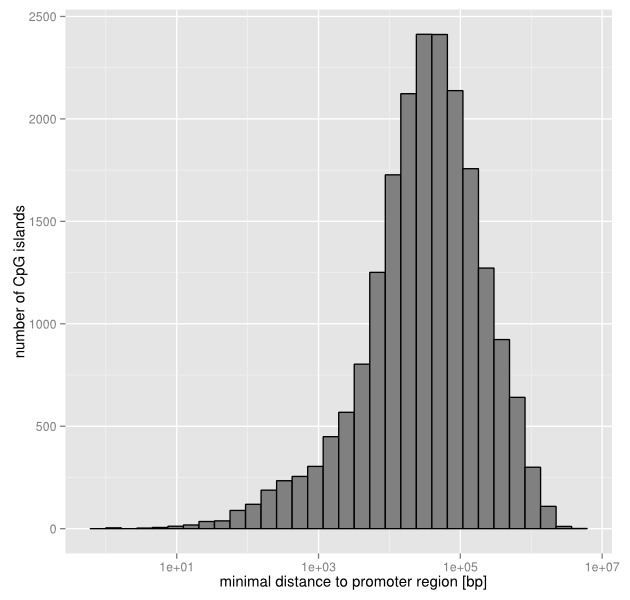


The histogram illustrates the spatial distance between promoter regions and CGIs (total number of CGIs outside of promoters = 22,036). We calculated it as the minimal distance between an annotated CGI and its closest annotated promoter (defined as 2kb upstream of TSS). The majority (78%) of CGIs was at least 10,000 nucleotides away from the next promoter. At such distance CGIs were not expected to have promoter-activity.
