# Supplementary material for: Whole genome sequencing and methylome analysis of the wild guinea pig
Source: BMC Genomics. 2014 Nov 28;15(1):1036. doi: 10.1186/1471-2164-15-1036 (PMC4302102; doi:10.1186/1471-2164-15-1036)
Supplement: Supplementary file 5 — Additional file 5: Table S3: Selection of imprinted genes and of non-imprinted genes regulated by methylation found by MEBS. Word document, named: Weyrich_BMC_AdditionalFiles_2014-11-03_resubmission. (DOC 41 KB) [file 12864_2014_6847_MOESM5_ESM.doc]

**Additional file 5: Table S3 - Selection of imprinted genes and of non-imprinted genes regulated by methylation found by MEBS**

| **Imprinted genes** | **Scaffold_No** | **Genomic localization** | **Average sequencing depth ^(1)^** | **Min-Max sequencing depth ^(2)^** | **Number and percentage of uncovered Cs^(3)^** |
| --- | --- | --- | --- | --- | --- |
| *Grb10* | scaff_102 | 2,241,042-2,276,285 | 3 | 0-46 | 9,666 (51.0%) |
| *Igf2^(4)^* | scaff_42 | 12,403,167-12,417,530 | 1 | 0-32 | 3,370 (77.0%) |
| *Kcnq1* | scaff_42 | 11,765,971-12,079,455 | 1 | 0-63 | 92,686 (84.0%) |
| *Peg10^(4)^* | scaff_11 | 2,6071,774-2,607,4167 | 5 | 0-63 | 329 (26.0%) |
| *Peg3* | scaff_175 | 714,792-725,778 | 4 | 0-79 | 2,977 (49.0%) |
| *Snrpn* | scaff_151 | 278,062-281,715 | 1 | 0-37 | 1,156 (75.0%) |
| *Snrpn* | scaff_123 | 2,621,848-2,623,149 | 2 | 0-18 | 328 (54.0%) |
| *Gnas* | scaff_156 | 1,121,751-1,174,547 | 1 | 0-26 | 14,347 (72.0%) |
|  |  |  |  |  |  |
| **Methylated genes** | **Scaffold_No** | **Genomic localization** | **Average sequencing depth ^(1)^** | **Min - Max sequencing depth ^(2)^** | **Number and percentage of uncovered Cs^(3)^** |
| *Bdnf^(4)^* | scaff_74 | 1,486,578-1,487,345 | 12 | 0-37 | 10 (2.0%) |
| *Hnf4a* | scaff_45 | 10,441,433-10,462,946 | 2 | 0-48 | 7,096 (72.0%) |
| *Ppara^(4)^* | scaff_160 | 1,928,325-1,971,506 | 6 | 0-93 | 6,980 (29.0%) |

The table lists selected imprinted and other methylated genes that were detected in the MEBS reads. ^(1)^ We calculated the average sequencing depth, as the sum of coverage for a gene`s Cs, divided by the total number of Cs within its genomic range. ^(2)^ We included minimal and maximal number of mapped MEBS reads per site in that genomic range as "min/max sequencing depth". Since MEBS reads didn’t cover the full gene, sequencing depth = 0 occurred at some positions. ^(3)^ Column shows the amount and the percentage of Cs that where not sequenced by MEBS, but known to be present from the `*C.aperea* reference sequence`. In every gene there are both Cs with high and no sequencing depth, which indicates the selection for methylated regions by MEBS. ^(4)^ Selected genes shown in figure 6.
